# Accuracy in Mixed Dentition Arch Length Discrepancy Prediction Methods in Surabayan Children

**DOI:** 10.1055/s-0045-1812496

**Published:** 2026-01-08

**Authors:** Alexander Patera Nugraha, Andree Salim, Delfia Amanda Putri, Ari Triwardhani, I Gusti Aju Wahju Ardani, Ida Bagus Narmada, Ervina Restiwulan Winoto, Alida Alida, Adya Pramusita, Ratna Nurlia Alfiandini, Tengku Natasha Eleena binti Tengku Ahmad Noor

**Affiliations:** 1 Department of Orthodontics, Faculty of Dental Medicine, Airlangga University, Surabaya, Indonesia; 2 Division for Globalization Initiative, Liaison Center for Innovative Dentistry, Graduate School of Dentistry, Tohoku University, Sendai, Japan; 3 Membership of the Faculty of Dental Surgery, The Royal College of Surgeons of Edinburgh, Edinburgh, United Kingdom; 4 Centre of Studies for Periodontology, Faculty of Dentistry, Universiti Teknologi MARA (UiTM), Sungai Buloh, Selangor, Malaysia

**Keywords:** arch length discrepancy, dentistry, malocclusion, medicine, orthodontics

## Abstract

**Objective:**

Accurate space evaluation in malocclusion using arch length discrepancy (ALD) analysis is essential for effective orthodontic interceptive treatment during the mixed dentition (MD) phase and growth and development stage (GDS). The study aimed to analyze ALD using the Sitepu, Moyers, and Tanaka– Johnston methods and also the ALD measurement technique (brass wire and segmentation technique) in Surabayan children with Angle's Class I malocclusion during the MD and GDS.

**Materials and Methods:**

A total of 60 study models of Javanese children during MD and GDS at the Dental Hospital of Airlangga University were used based on the inclusion criteria. Cervical vertebral maturation stage was used to determine the GDS. Lateral cephalometry was investigated with Steiner's analysis < A point–nasion–B point to determine the skeletal malocclusion. ALD analysis was conducted based on the Moyers, Sitepu, and Tanaka–Johnston methods.

**Results:**

There was significant difference in ALD for the maxillary arch ( *p* < 0.05), while no significant difference was found in the mandibular arch ( *p* > 0.05). There were significant differences between the Moyers and Sitepu methods in the maxillary arch ( *p* < 0.05), but no significant difference between the Moyers and Tanaka–Johnston or Sitepu and Tanaka–Johnston methods ( *p* > 0.05). There was a significant difference between the Moyers segmented and brass wire methods in the maxilla ( *p* < 0.05), with no significant difference in the mandible ( *p* > 0.05).

**Conclusion:**

The results of this MD and GDS study showed there were statistically significant differences in ALD among the Moyers, Sitepu, and Tanaka–Johnston methods for the maxilla, but not the mandibular arches.

## Introduction

Malocclusion is an issue found in dentofacial development and is commonly observed during the mixed dentition (MD) period, which is a transitional phase from primary teeth to permanent teeth. 1 Dental malocclusion is primarily caused by a mismatch between the dental arch length and tooth size. 2 Based on Angle's classification of the relationship between the maxillary and mandibular first molars, malocclusion is divided into Classes I, II, and III. Angle's Class I malocclusion is the most frequently encountered case. 3 Many factors contribute to malocclusion, including hereditary and environmental aspects. 4 The prevalence of malocclusion in Indonesia remains very high, affecting approximately 80% of the total population and representing a major dental health issue. If orthodontic treatment is required, it should begin during the MD stage, as this is the optimal time to prevent malocclusion. 5 6 7

Orthodontic treatment often requires adequate space to ensure proper alignment of teeth within a stable arch. Evaluating space requirements involves specific analyses to determine the appropriate treatment method. One such analysis is arch length discrepancy (ALD). ALD refers to the difference between available space (AS) and required space (RS). AS is defined as the arch length available for tooth placement, while RS represents the total mesiodistal width of the teeth from the left second premolar to the right second premolar in each arch. 8 ALD analysis for space requirements includes several methods, such as the Moyers, Sitepu, and Tanaka–Johnston methods.

The Moyers method utilizes probability tables to estimate the space needed for predicting permanent canine (C), first premolar (P1), and second premolar (P2) (C-P1-P2) in the maxilla and mandible using the sum of the four mandibular permanent incisors as a predictor. Moyers employs prediction tables at the 75th percentile, as it is considered globally reliable and safe against malocclusion. The Sitepu method typically analyzes space requirements by summing the widths of the four mandibular incisors and applying a formula. Although the Sitepu method may be accurate, its reliability may be compromised by measurement errors. 1 The Tanaka–Johnston method references the four mandibular permanent incisors and establishes a predictive equation based on formulas and constants applied to each arch. This method is simple and convenient, as it does not require radiographic imaging. 9

Malocclusion is significantly associated with the most prevalent dental illnesses in children, including dental caries, pulpal and periapical lesions, dental trauma, abnormalities of development, and oral habits. Pediatric dental clinics manage oral health in the early infancy stage to reduce the unintended impact of these conditions on dentition. 10 Age determination is crucial in many professions, especially in orthodontics. An accurate way to evaluate a person's development and progress is to look at their biological and chronological age. By looking at skeletal or dental maturation, one might ascertain biological age. Precisely determining growth potential and growth spurt timing is essential in many therapeutic settings, particularly when it comes to treatment planning and results, such as interruptive orthodontic treatment during growth and development. 11 The shape and size of the dental arch are essential components of orthodontic diagnosis and treatment planning. Its size will grow as a result of tooth eruption, and it is also influenced by gender, systemic illness, diet, ethnicity, and hormones. Children between the ages of 8 and 10 years have grown several permanent teeth. 12 Furthermore, soft tissue morphology, development direction and pattern, malocclusion categorization, incisive axial inclination correlations, jaw bone–base interactions, face skeletal structures, and orthodontic treatment limits are all revealed by the examination of cephalometric radiographs. Skeletal malocclusion classification is determined based on sella–nasion–A point (SNA) minus sella–nasion–B point (SNB), known as A point–nasion–B point (ANB). 13

ALD analysis measures space requirements by comparing the arch length to the dental arch length. The Moyers, Sitepu, and Tanaka–Johnston methods share similarities in evaluating space requirements by comparing the four permanent incisors. However, the study that compares the accuracy of Moyers, Sitepu, and Tanaka–Johnston, and also the ALD measurement technique (brass wire and segmentation technique) in Surabayan children with MD phase during growth and development stage (GDS) is still limited. Furthermore, the hypothesis of this study regarding ALD calculated from different formulas as well as the technique for actual arch length measurement may be significantly different. Therefore, this study is interested in conducting an analysis study of ALD using the Sitepu, Moyers, and Tanaka–Johnston methods, and also the ALD measurement technique (brass wire and segmentation technique) in Surabayan children with Angle's Class I malocclusion during the MD and GDS with Angle's Class I malocclusion at the Dental Hospital, Universitas Airlangga (UNAIR) in 2023 to 2024.

## Materials and Methods

### Study Design and Ethical Clearance

This study adopted a cross-sectional design, involving a total population of 253 patients from the Department of Orthodontics at the Dental Hospital, Universitas Airlangga, Surabaya. The research was conducted between July and November 2024, focusing on children aged 7 to 12 years who had either received or not yet received orthodontic treatment. Data collection included orthopantomography, lateral cephalograms, and study models obtained from orthodontic patients treated between 2023 and 2024. Ethical approval for the study was granted by the Health Research Ethics Committee, Faculty of Dental Medicine, Universitas Airlangga (35/UN3.9.3/Etik/PT/2024) with date approval August 14, 2024.

**Fig. 1:**
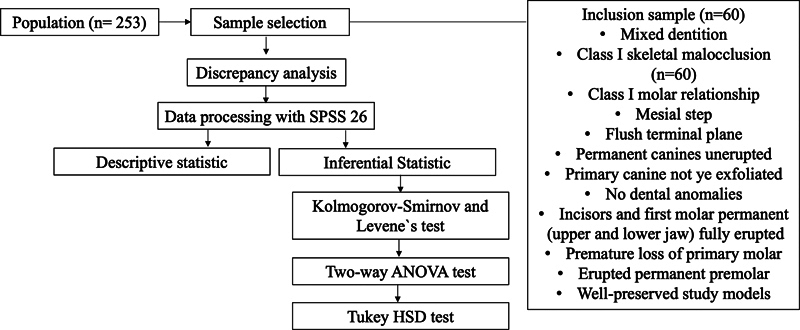
Sample selection process based on inclusion and exclusion criteria.

From the total population using the total sampling method, 60 samples were selected based on specific inclusion criteria. The criteria required participants to have skeletal Class I malocclusion, a Class I molar relationship, or an edge-to-edge molar relationship with the second primary molar in a flush terminal plane or mesial step position. Additionally, the first permanent molars had to be fully erupted, as well as the central and lateral permanent incisors in all dental arches. Dental arch development in terms of dental stage known as “early mixed dentition” represents the period when the permanent canines and premolars have not yet erupted. Samples were excluded if they exhibited erupted permanent canines, agenesis of permanent canines or premolars, premature loss of primary canines, congenital anomalies, or dental deformities such as macrodontia or microdontia (see Fig. 1 ).

**Fig. 2:**
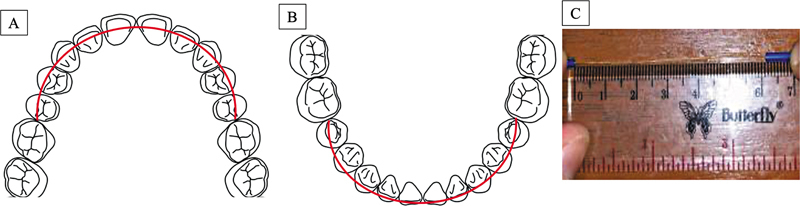
Brass wire technique. ( **A** ) Upper arch, ( **B** ) lower arch, and ( **C** ) measuring with a ruler.

**Fig. 3:**
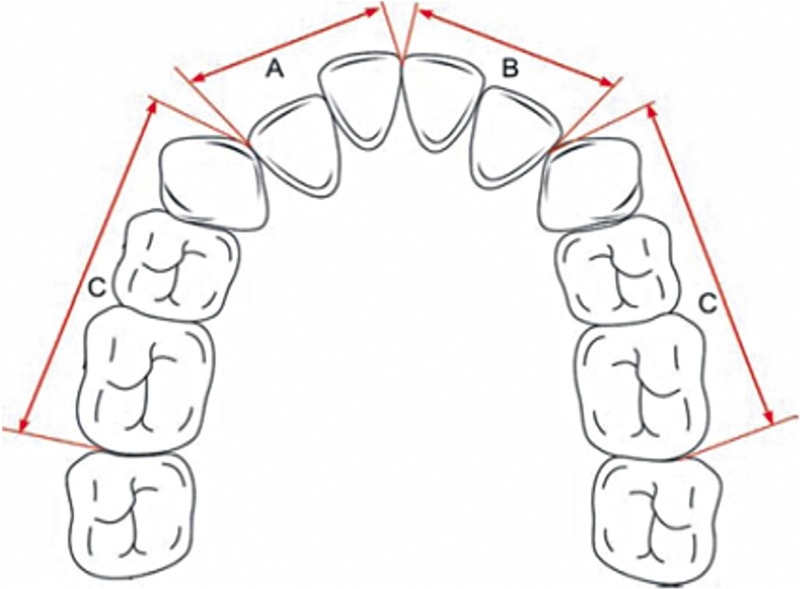
Division of the dental arch into four segments with segmentation method.

The discrepancy value is determined by subtracting the RS from the AS. The calculation of AS was conducted using two methods: the brass wire technique and the segmentation technique. For the brass wire technique, this study utilized a 0.5-mm brass wire. The process involves positioning the brass wire along the dental arch, starting from the mesiobuccal cusp of the first permanent molar on one side and extending to the mesiobuccal cusp of the molar on the opposite side. The wire (red color) follows the buccal contour of the lower arch (see Fig. 2A ) or the external fissure of the upper arch (see Fig. 2B ) and aligns with the incisal edges of the teeth to create the appropriate curvature (red color). Once positioned, the wire is straightened, and its length is measured using a standard ruler (see Fig. 2C ). In the segmentation technique (see Fig. 3 ), each jaw is divided into four segments, beginning with M2 to the deciduous canine, lateral incisor to central incisor, central incisor to lateral incisor, and deciduous canine to M2. The values of all segments are then added together to determine the total AS.

#### Moyers Method

In this study, the 75% probability table of the Moyers method (see Table 1 ) was used to estimate the width of the permanent canine and premolar teeth based on the mesiodistal width of the lower permanent incisors. The calculation involves measuring the mesiodistal width of the four lower incisors and adding the corresponding value from the 75% probability table twice. The predicted values are then compared with the available dental arch length. If the predicted value exceeds the available length, crowding occurs; if it is smaller, spacing will result.

**Table 1:** Moyers prediction table with 75% probability 24

**Mandibular bicuspid and cuspid**													
21|12	19.5	20.0	20.5	21.0	21.5	22.0	22.5	23.0	23.5	24.0	24.5	25.0	25.5
Males 75%	20.3	20.5	20.8	21.0	21.3	21.5	21.8	22.0	22.3	22.5	22.8	23.0	23.3
Females 75%	19.6	19.8	20.1	20.3	20.6	20.8	21.1	21.3	21.6	21.9	22.1	22.4	22.7
**Maxillary bicuspid and cuspid**													
21|12	19.5	20.0	20.5	21.0	21.5	22.0	22.5	23.0	23.5	24.0	24.5	25.0	25.5
Males 75%	20.3	20.5	20.8	21.0	21.3	21.5	21.8	22.0	22.3	22.5	22.8	23.0	23.3
Females 75%	20.4	20.5	20.6	20.8	20.9	21.0	21.2	21.3	21.5	21.6	21.8	21.9	22.1

The Moyers formula to predict the RS in the maxilla and mandible is as follows:

Required space = sum of upper incisors + (2 × predicted value of the maxilla)

Required space = sum of lower incisors + (2 × predicted value of the mandible)

#### Sitepu Method

In the measurement using the Sitepu method, the mesiodistal width of the four mandibular incisors is measured and then summed. This total is compared with the Sitepu prediction table (see Table 2 ), resulting in the mesiodistal width values of the second premolar, first premolar, and canine on one side of the maxilla (R.A) or mandible (R.B).

**Table 2:** Sitepu prediction table 25

X	Y R.A	Y R.B
19.5	21.16	19.88
20.0	21.40	20.11
20.5	21.64	20.34
21.0	21.88	20.57
21.5	22.12	20.80
22.0	22.37	21.03
22.5	22.61	21.26
23.0	22.85	21.49
23.5	23.09	21.72
24.0	23.34	21.95
24.5	23.58	22.18
25.0	23.82	22.41
25.5	24.06	22.64
26.0	24.30	22.87
26.5	24.55	23.10
27.0	24.79	23.33
27.5	25.03	23.56
28.0	25.27	23.79
28.5	25.51	24.02
29.0	25.76	24.25

The Sitepu formula to predict the RS in the maxilla (R.A) and mandible (R.B) is as follows:

The required space = total width of the upper incisors + (2 × Y R.A)

The required space = total width of the lower incisors + (2 × Y R.B)

The Y R.B value is the total mesiodistal width of the second premolar, first premolar, and canine on one side of the mandible. Meanwhile, the Y R.A value is the total mesiodistal width of the second premolar, first premolar, and canine on one side of the maxilla. 14

#### Tanaka–Johnston Method

The Tanaka–Johnston method to predict the RS in the maxilla and mandible uses the following formula:





The required space = total width of the upper incisors + (2 × { + 11 mm)

The first step in determining the predicted value based on the Tanaka–Johnston method is to calculate the mesiodistal width of the four mandibular incisors. The predictive value for unerupted one side maxillary canine and two premolars is “(sum of mesiodistal widths of four lower incisors/2) + 11.0 mm.” Similarly, for mandibular unerupted canine and two premolars on one side is “(sum of mesiodistal widths of four lower incisors/2) + 10.5 mm” to obtain the estimated width of the maxillary canine and premolars in one quadrant. 15

### CVMS Method

The cervical vertebral maturation stage (CVMS) method by Baccetti is a simple and commonly used visual method that focuses on the second, third, and fourth cervical vertebrae. 16 The first step in analyzing cervical vertebral maturation using the CVMS method is to prepare cephalometric photos, tracing paper, pencils, erasers, and rulers. Then, redraw the radiographic image by tracing the inferior border and the shape of the second (C2), third (C3), and fourth (C4) cervical vertebral bodies. Then, match the tracing results with the CVMS characteristic table to determine the interpretation of the patient's skeletal maturity level.

### Steiner Cephalogram Analysis

Skeletal malocclusion is often determined cephalometrically by comparing the relationship between the maxilla and mandible with the cranial base using the SNA, SNB, and ANB angles. This analysis is performed using the WebCeph automated Cephalometry tracing software ( https://webceph.com/ , WEBCEPH version 2.0.0, Dental Imaging Software, AssembleCircle Corp., Gyeonggi-do, Republic of Korea).

### Statistical Analysis

All measurements were carried out by double-blinded operators with previously calibrated to ensure high precision, reliability, and accuracy. Statistical analysis was conducted on 60 samples that met the inclusion criteria using Statistical Package for Social Science (SPSS) version. 25 (IBM corporation, Chicago, Illinois, United States), with a significance level set at *p* < 0.05. Descriptive analysis was performed to assess the distribution and frequency of the samples. The results of the Kolmogorov–Smirnov's test and Levene's test confirmed that the data followed a normal distribution and exhibited homogeneity. Therefore, further analysis was performed using the parametric two-way analysis of variance (ANOVA) and Tukey's honest significant difference (HSD) test to determine significant differences in the cumulative data from the Moyers, Sitepu, and Tanaka–Johnston methods.

## Results

A total of 60 samples met the inclusion criteria, with a higher proportion of males than females (51.7%). The average chronological age was 9 years, with a relatively narrow age range despite some variations. Skeletal malocclusion was analyzed using the SNA, SNB, and ANB angles through WebCeph automated cephalo tracing software. The SNA values ranged from 82.16 to 84.82 degrees, with an average of 83.49 degrees, indicating an orthognathic maxilla. The SNB values ranged from 79.65 to 82.22 degrees, with an average of 80.93 degrees, representing an orthognathic mandible. Meanwhile, the ANB values ranged from 2.20 to 2.89 degrees, with an average of 2.55 degrees, indicating skeletal Class I. All samples were classified as skeletal Class I malocclusion, characterized by a straight facial profile and a harmonious relationship between the upper and lower arches (see Table 2 ). Based on the analysis of the CVMS, all samples have a skeletal age in the prepubertal phase, corresponding to CVMS stage 1 and stage 2 (see Table 3 ).

**Table 3:** Descriptive statistical analysis of sex, bone age, chronological age, <SNA, <SNB, <ANB

Parameters	Frequency	Percentage
Sex		
Boy	31	51.7
Girl	29	48.3
Bone age		
CVMS 1	40	66.7
CVMS 2	20	33.3
**Parameters**	**Mean ± SD**	
Chronological age	9.00 ± 0.883	
SNA	83.49 ± 5.15 deg	
SNB	80.93 ± 4.97 deg	
ANB	2.55 ± 1.33 deg	

Abbreviations: ANB, A point–nasion–B point; CVMS, cervical vertebral maturation stage; SD, standard deviation; SNA, sella–nasion–A point; SNB, sella–nasion–B point.

For the space required, the Moyers method has a lower mean but higher standard deviation compared with the Sitepu and Tanaka–Johnston methods in the upper arch, indicating less consistent measurements. In the lower arch, the Moyers, Sitepu, and Tanaka–Johnston methods show relatively similar mean values and standard deviations. Regarding the space available, the brass wire technique in the upper arch shows a higher mean and greater variability, while in the lower arch, it has a lower mean with higher variability compared with the segmental technique. These findings indicate minor differences in the average space available between the two techniques in both jaws (see Table 4 ).

**Table 4:** Descriptive statistical analysis of space available and space required value

	*N*	Mean ± SD
Space available		
Upper arch		
Brass wire technique	60	68.59 ± 3.07
Segmented technique	60	68.47 ± 2.89
Lower arch		
Brass wire technique	60	65.28 ± 3.12
Segmented technique	60	65.76 ± 2.96
Space required		
Upper arch		
Moyers	60	66.16 ± 2.85
Sitepu	60	68.50 ± 2.55
Tanaka–Johnston	60	66.86 ± 1.97
Lower arch		
Moyers	60	65.98 ± 2.90
Sitepu	60	65.78 ± 2.49
Tanaka–Johnston	60	66.82 ± 2.63

Abbreviation: SD, standard deviation.

In the upper arch, the average discrepancy values for the Moyers and Tanaka–Johnston methods were positive, while the Sitepu method showed a negative value. In the lower arch, the average discrepancy values for the Moyers, Sitepu, and Tanaka–Johnston methods were negative, with the Tanaka–Johnston method showing the lowest average value (see Table 5 ). The results of the Shapiro–Wilk's normality test and Levene's homogeneity test indicated that the data met the assumptions of normality and homogeneity ( *p* < 0.05) (see Table 6 ). The results of the independent sample *t* -test on discrepancy values in both jaws using the Moyers brass wire and segmented methods showed a significant difference in the upper arch ( *p* < 0.05). However, no significant difference was observed in the lower arch ( *p* > 0.05) (see Table 7 ). The results of the one-way ANOVA test on the upper arch discrepancy data showed a significant difference among the three methods ( *p* < 0.05), using both the segmented and brass wire techniques. However, no significant difference was found in the lower arch discrepancy data ( Table 8 ). The results of the Tukey's HSD test on the upper arch discrepancy data showed a significant difference between the Moyers and Sitepu methods, using both the segmented and brass wire techniques. However, no significant differences were found between the Sitepu and Tanaka–Johnston methods or between the Moyers and Tanaka–Johnston methods with either the segmented or brass wire techniques. In the lower arch, no significant differences were observed among the three methods using either the segmented or brass wire techniques (see Table 9 ). In the segmentation technique (see Fig. 3 ), each jaw is divided into four segments, beginning with M2 to the deciduous canine, lateral incisor to central incisor, central incisor to lateral incisor, and deciduous canine to M2. The values of all segments are then added together to determine the total AS.

**Table 5:** Descriptive statistical analysis of ALD value in both arches

Methods	*N*	Mean ± SD
Upper arch		
Moyers brass wire	60	2.32 ± 1.41
Moyers segmented	60	2.3 ± 1.39
Sitepu brass wire	60	0.09 ± 1.58
Sitepu segmented	60	−0.03 ± 1.53
Tanaka–Johnston brass wire	60	1.73 ± 1.73
Tanaka–Johnston segmented	60	1.60 ± 1.61
Lower arch		
Moyers brass wire	60	−0.9 ± 1.84
Moyers segmented	60	−0.43 ± 1.98
Sitepu brass wire	60	−0.5 ± 2.08
Sitepu segmented	60	−0.021 ± 2.06
Tanaka–Johnston brass wire	60	−1.54 ± 2.12
Tanaka–Johnston segmented	60	−1.05 ± 2.10

Abbreviations: ALD, arch length discrepancy; SD, standard deviation.

**Table 6:** Result of normality and homogeneity test of ALD examined by means of Moyers, Sitepu, and Tanaka–Johnston methods

Methods	*N*	Significance (Kolmogorov–Smirnov) a	Significance (Levene's test)
Upper arch			
Moyers brass wire	60	0.200	0.958
Moyers segmented	60	0.200	
Sitepu brass wire	60	0.200	
Sitepu segmented	60	0.200	
Tanaka–Johnston brass wire	60	0.200	
Tanaka–Johnston segmented	60	0.200	
Lower arch			
Moyers brass wire	60	0.200	0.828
Moyers segmented	60	0.200	
Sitepu brass wire	60	0.200	
Sitepu segmented	60	0.200	
Tanaka–Johnston brass wire	60	0.200	
Tanaka–Johnston segmented	60	0.200	

Abbreviation: ALD, arch length discrepancy.

a Normal and homogeneity at *p* > 0.05.

**Table 7:** Results of independent sample *t* -test for ALD examined by means of Moyers method

	Significance
Moyers UA brass wire	0.025 a
Moyers UA segmented	
Moyers LA brass wire	0.054
Moyers LA segmented	

Abbreviations: ALD, arch length discrepancy; LA, lower arch; UA, upper arch.

a Significant difference at *p* < 0.05.

**Table 8:** Result of the two-way ANOVA test for ALD examined by means of Moyers, Sitepu, and Tanaka–Johnston methods

ANOVA		Significance
Brass wire UA	Between groups	0.048 a
Brass wire LA	Between groups	0.543
Segmented UA	Between groups	0.007 a
Segmented LA	Between groups	0.368

Abbreviations: ALD, arch length discrepancy; ANOVA, analysis of variance; LA, lower arch; UA, upper arch.

a Significant difference at *p* < 0.05.

**Table 9:** Result of the Tukey's HSD test for ALD examined by means of Moyers, Sitepu, and Tanaka–Johnston methods

Dependent variable	Group	Group	Significance
Brass wire UA	Sitepu	Moyers	0.037 a
		Tanaka	0.355
	Moyers	Tanaka	0.51
Segmented UA	Sitepu	Moyers	0.005 a
		Tanaka	0.177
	Moyers	Tanaka	0.338

Abbreviations: ALD, arch length discrepancy; HSD, honest significant difference; UA, upper arch.

a significant different between groups at *p* < 0.05.

## Discussion

In the current study, Surabayan children with Angle's Class I malocclusion were examined for ALD employing the Sitepu, Moyers, and Tanaka–Johnston methodologies as well as ALD measuring techniques (brass wire and segmentation technique) during the MD and GDS. The study comparing the Moyers method using brass wire and segmented approaches on 60 study models at Rumah Sakit Gigi dan Mulut Pendidikan (RSGMP) UNAIR showed a significant difference in measurements for the maxilla ( *p* < 0.05). Johal and Battagel in 1997 found a 6-mm difference in measurements between the brass wire and segmented methods in orthodontic patients. 17 Similarly, Wang et al in 2023 reported a difference of 3.44 mm. According to Wang et al, the segmented method, using calipers, produces significantly lower estimates of tooth space deficiency compared with the brass wire method. Brass wire relies on the operator's subjective assessment to create an ideal dental arch, leading to inconsistent and less reproducible results. In contrast, the segmented method simplifies the dental arch into straight-line segments, often underestimating the actual arch circumference. 18

The study comparing the three ALD methods, Moyers, Sitepu, and Tanaka–Johnston, presented that for brass wire measurements on the maxilla, there is a significant difference among the three methods ( *p* < 0.05), while no significant difference is observed for the mandible. Contrastingly, Puri et al in 2022 found that ALD comparisons using these methods on study models of 8 to 12-year-old students at National Elementary School or *Sekolah Dasar Negeri* Gubeng 1 revealed significant differences in the mandible ( *p* < 0.05) but not in the maxilla. 5 The difference in findings may stem from the higher accuracy of mandibular incisors compared with maxillary lateral incisors, which exhibit greater size variation. 19 Other contributing factors include racial differences, as the Moyers and Tanaka–Johnston methods were developed based on Caucasian children in North America, while the Sitepu method is based on the Deutero-Malay population. 5 Environmental influences such as dietary habits and oral behaviors, genetic factors, and interpopulation tooth size variations also affect the results of ALD analyses across the three methods. 20

The comparison of ALD analysis methods using brass wire on 60 study models at RSGMP UNAIR showed a significant difference between the Moyers and Sitepu methods for the maxilla ( *p* < 0.05). This aligns with Kaswindiarti and Widayati in 2022, who found similar differences in children at Islamic Junior High School or *Sekolah Menengah Pertama* (SMP) Al Islam 1 Surakarta, 1 as the Sitepu method is based on Deutero-Malay samples, while the Moyers method is based on Caucasian samples. 21 Higher variation in maxillary teeth, particularly first permanent molars and central and lateral permanent incisors, further explains the differing results between the two methods. 14

The comparison between the Sitepu and Tanaka–Johnston methods mentioned that there was no significant difference. This aligns with Kurniawan's 2006 study on Dental Medicine Universitas Airlangga students, which found that the Sitepu method was more suitable for predicting the mesiodistal width of canines and premolars in the Deutero-Malay population. However, the differences between the two methods were not statistically significant, indicating comparable accuracy in this population. 22 While the Tanaka–Johnston method was developed for Caucasian populations and the Sitepu method for Deutero-Malay populations, their similar results here can be attributed to the study's single-population focus with comparable dental characteristics. Both methods rely on linear measurements of erupted mandibular incisors, contributing to their comparable estimations. This present study also found that there was no significant difference between the Moyers and Tanaka–Johnston methods, consistent with Ravinthar and Gurunathan's 2020 study at Saveetha Hospital, which reported similar findings. 23 Doda et al in 2021 similarly found no significant difference between these methods, with both yielding overestimated results for the maxilla and mandible. 9 This lack of significant difference may be due to Tanaka–Johnston being a variation of the Moyers method, with its estimates for unerupted canine and premolar widths aligning with the 75% probability values in Moyers prediction table. 15 24 25 Both methods were developed using similar data from Caucasian populations and rely on highly correlated variables, resulting in nearly identical predictive outcomes. 20

There are several factors affecting ALD calculation, such as, arch length measurement (both actual and required arch length), incisor position, and degree of crowding. Based on this present study's results, it is quite challenging to determine which is the best formula for predicting the ALD in Surabayan children during MD and GDS due to the variation and uniqueness of morphologic characteristics. Thus, the limitation of this study is not only about increasing the sample size to enhance the accuracy, reliability, and precision to examine the ALD but also about developing a population-specific formula for determining arch length–tooth material discrepancy with more accurate results than the Moyers, Sitepu, and Tanaka–Johnston, which is especially directly applicable to Surabayan children.

## Conclusion

The results of this study revealed that the ALD analysis using the Moyers, Sitepu, and Tanaka–Johnston methods for Angle's Class I malocclusion in Surabayan children during the MD period and GDS at Universitas Airlangga Dental Hospital in 2023 to 2024 showed there were significant differences in ALD Moyers, Sitepu, and Tanaka–Johnston methods for the maxilla but not for the mandible. Further research with a larger sample population is necessary to enhance the validity and reliability of the conclusions. It is also necessary to create a population-specific formula that is specifically relevant to Surabayan children and yields more accurate findings than the Moyers, Sitepu, and Tanaka–Johnston formulas for determining the arch length–tooth material mismatch.
